# The association of zero walking cadence minutes with sleep quality in adults 18–65

**DOI:** 10.1007/s11325-024-03175-1

**Published:** 2024-11-25

**Authors:** Trent A. Hargens, Matthew C. Scott, Meghan Peterson, Kimberly C. Bennett, Timothy Thome, Elizabeth S. Edwards

**Affiliations:** 1 Human Performance Laboratory, Integrative Nutrition and Physiology Laboratory, Department of Kinesiology MSC 2302, James Madison University, 261 Bluestone Dr., Harrisonburg, VA 22807 USA; 2 Department of Kinesiology and Health Sciences, Virginia Commonwealth University, Richmond, VA 23284 USA; 3 Human Performance Laboratory, Department of Kinesiology, James Madison University, Harrisonburg, VA 22807 USA; 4 Human Performance Laboratory, Morrison Bruce Center, Department of Kinesiology, James Madison University, Harrisonburg, VA 22807 USA

**Keywords:** Step cadence, Sedentary time, Physical activity, Zero cadence, Sleep efficiency

## Abstract

**Purpose:**

Physical activity (PA) guidelines suggest a link between PA, sedentary time, and sleep quality (SQ). Step cadence is an emerging proxy for PA intensity, with zero cadence (ZC) indicating sedentary time. No research has explored the relationship between ZC and SQ. This study examines the relationship between ZC and SC, compared to traditional PA and sedentary metrics, all measured via accelerometry.

**Methods:**

Sleep and PA was assessed in 138 adults (53 male, 85 female, age = 23.5 ± 8.0 year.) via accelerometry. Sedentary, light, moderate, vigorous intensity PA, and ZC minutes per day were measured. SQ variables included sleep efficiency (SE), total sleep time (TST), and minutes of awake time after sleep onset (WASO). Correlation analysis and analysis of covariance was used to assess relationships between study variables and to assess differences in PA and sedentary behavior between normal and poor sleepers.

**Results:**

Sedentary time was negatively associated with SE (*r* = -0.24, *P* < 0.05) and TST (*r* = -0.51, *P* < 0.001). ZC was negatively associated with SE (*r* = -0.25, *P* < 0.05), TST (*r* = -0.39, *P* < 0.001) and positively associated with WASO (*r* = 0.17, *P* < 0.001). Poor sleepers accumulated greater ZC minutes than normal sleepers when categorized by SE (555.9 ± 69.1 vs. 521.6 ± 83.7, *P* = 0.02)) or TST (570.4 ± 77.7 vs. 524.5 ± 76.7, *P* < 0.01).

**Conclusion:**

Results suggest that zero cadence minutes is a viable metric for assessing sedentary time and may be of greater utility to more traditional measure of sedentary time.

## Introduction

Published in 2018, the Physical Activity Guidelines for Americans concludes that increases in moderate-to-vigorous intensity physical activity (MVPA) improves the quality of sleep in adults [1]. In addition, it is recommended that all adults “move more and sit less”, thereby reducing the time spent daily in sedentary time [1]. Sedentary time is defined as any waking behavior with an energy expenditure less than or equal to 1.5 metabolic equivalents (METs), typically while sitting, reclining or lying [1]. The Physical Activity Guidelines for Americans characterizes standing as distinct from sedentary behavior, but has also characterized sedentary by low levels of movement measured by devices that assess movement or posture [1].

Cadence (steps/min) is a variable that has traditionally been measured in short distance walking tests, but has increasingly been used as marker of physical activity (PA) intensity due to its relationship to speed, making it a useful surrogate variable of intensity [2, 3]. With the advent of accelerometry, as well as consumer wearables that track steps, cadence is a measure becoming increasingly available to the general public. Minute-by-minute cadence data is now possible to assess, including time spent not taking steps. Zero steps taken in a given minute represents “non-movement” and can include taking no steps while standing, which contrasts the more traditional definition of sedentary time defined above [2]. Additionally, traditional sedentary time utilizes the absolute intensity criteria of 1.5 METs across the population, regardless of the functional capacity of the individual. For an individual with a maximal functional capacity of 4 METs, 1.5 METs represents 26% of their functional capacity. For an individual with a 15 MET capacity, 1.5 METs is 10% of their functional capacity. Therefore, PA at 1.5 METs intensity represents a very different intensity for these two individuals. Zero cadence (ZC), however, more directly assesses non-movement, which may better capture true sedentary time across populations. Despite its potential, little published research has examined minutes of zero step cadence as a surrogate marker for sedentary behavior [4–6], and to date, no studies have examined the relationship between zero cadence minutes and sleep quality (SQ).

Research simultaneously examining objectively measured PA, SQ, and sedentary behavior patterns remains limited [7–12]. In three of these studies, middle-aged and older Japanese adults were studied, and utilized self-report questionnaire data for SQ, and accelerometer assessment for PA and sedentary time, using the traditional definition of sedentary time (< 1.5 METs) [9, 10, 12]. One study used objective accelerometer data for sedentary time and SQ and found a negative relationship between the two in a sample of college students in China [11]. This study did not, however, assess the relationship to PA. One study used objective accelerometer data for PA, sedentary time, and SQ simultaneously in a sample of college students in the United States [11]. Hargens et al. found that sedentary behavior was more strongly associated with poor sleep than PA [7]. Furthermore, poor sleep, as measured by total sleep time (TST), was negatively associated with the number of sedentary minutes accumulated the following day, for all days of the week [7]. Given these findings, it becomes essential to further explore how newer metrics, like ZC might offer additional insights into the relationship between PA, sedentary time, and SQ. Therefore, the purpose of this study is to examine the relationship between SQ, traditional measures of PA and sedentary behavior, and ZC minutes, all objectively measured through accelerometry. This investigation will provide new insights into how zero cadence, as a direct marker of non-movement, relates to SQ and PA behavior, building on existing research that emphasizes the impact of sedentary time on health.

## Methods

### Procedures

Adults between the ages of 18 and 65 were recruited from the campus of James Madison University (JMU) and surrounding community through bulk email communications, flyers, and word-of-mouth. Exclusion criteria included current smoker, prior diagnosis of cardiovascular, pulmonary, or metabolic disease, and limits to mobility. Ultimately, 138 subjects (53 male, 85 female) were recruited for this study (mean age 23.5 ± 8.5 years). A more detailed summary of subject characteristics is presented in Table 1. All research procedures were approved by the Institutional Review Board of JMU. Subjects completed an informed consent document prior to participation. Subjects completed two visits to the JMU Human Performance Laboratory. Visit 1 consisted of height and weight assessment and instructions on proper wear and use of the accelerometer for PA and sleep assessment. Height was measured with a wall-mounted stadiometer to the nearest 0.5 cm. Weight was measured to 0.1 kg on a digital scale with minimal clothing and no shoes. Visit 2 was to return the accelerometer.

**Table 1:** Subject characteristics. Data presented ± SD. WASO = wake after sleep onset, PA = physical activity, MVPA = moderate-to-vigorous intensity physical activity *P < 0.05 vs. males

	All (*n* = 138)	Male (*n* = 53)	Female (*n* = 85)
Age (years)	23.5 ± 8.5	24.8 ± 10.3	22.7 ± 7.1
Height (cm)	170.2 ± 8.8	177.0 ± 7.5	166.0 ± 6.6*
Weight (kg)	76.2 ± 18.1	84.9 ± 19.3	70.9 ± 15.0*
BMI (kg/m^2^)	25.1 ± 4.6	26.3 ± 4.9	24.3 ± 4.3
Days assessed	7.4 ± 1.3	7.6 ± 1.3	7.3 ± 1.3
Wear time (hours)	16.4 ± 1.9	16.4 ± 2.1	16.4 ± 1.8
Sleep latency (min)	7.1 ± 5.6	6.8 ± 4.0	7.4 ± 6.4
Sleep Efficiency (%)	85.3 ± 6.2	84.1 ± 6.1	86.0 ± 6.1*
Total sleep time (min)	398.0 ± 57.3	384.5 ± 59.8	406.5 ± 54.4*
WASO (min)	62.4 ± 29.1	66.9 ± 30.2	59.6 ± 28.3
Awakenings/night	19.4 ± 6.7	20.0 ± 7.2	19.0 ± 6.4
Length of awakenings (min)	3.3 ± 1.3	3.4 ± 1.2	3.2 ± 1.3
Sedentary time/day (min)	665.2 ± 79.0	676.8 ± 71.6	658.1 ± 82.9
Light intensity PA/day (min)	235.7 ± 70.2	230.3 ± 83.1	239.0 ± 61.0
MVPA/day (min)	59.4 ± 25.7	62.1 ± 30.6	57.8 ± 22.1
Steps/day	9343.3 ± 3276.1	9511.1 ± 4112.3	9238.8 ± 2647.0
0 cadence minutes/day	536.5 ± 79.3	546.0 ± 76.8	530.6 ± 80.7

### Instruments

Sleep, sedentary time, and PA were assessed via accelerometer (ActiGraph GT3X, ActiGraph Corp., Pensacola FL). A single accelerometer was used for each subject. Subjects were instructed to wear the accelerometer at the waist level in line with the right anterior axillary line during all waking hours, excluding any water-based activity. Subjects were instructed to wear the device for a minimum of 10 h per day but encouraged to wear it as much as possible while awake to assess sedentary and PA behavior. Accelerometer data was obtained in 1-minute epochs at a sampling rate of 30 Hz to obtain PA and sedentary data. These procedures are similar to the procedures utilized in the National Health and Nutrition Examination Survey (NHANES), which utilizes a minimum of 8 h of wear time per day [13]. To assess sleep, subjects were instructed to move the accelerometer from the waist position to the wrist of their non-dominant hand using a watch-style strap. Wrist-based accelerometry has been utilized in sleep research since 1982 [14, 15]. The algorithms used by the ActiGraph to detect sleep and wake periods have been extensively studied and provide comparable results to that of polysomnography [14–17]. Subjects completed a sleep log, documenting the specific time they attempt to fall asleep as well as the specific time of waking. Upon waking, subjects were instructed to move the accelerometer back to the waist position as soon as possible.

### Measurements

Accelerometer data was downloaded and analyzed with ActiLife software (ActiGraph Corp., Pensacola, FL). Wear time was determined according to methods developed by Choi et al. [18]. Cut points for PA intensity were those established by Freedson et al. [19]. A more detailed explanation of the cut points for PA intensity, can be found here [7]. From this, PA data obtained was light intensity minutes of PA per day, moderate-to-vigorous intensity minutes of PA per day, and steps per day. Sedentary minutes were defined as activity < 1.5 METs. To obtain ZC minutes per day, steps measured each day by the ActiGraph were stored in 1-minute epochs and were rank ordered for each day. The number of epochs in which the step count equaled 0 were counted for each day, and then averaged across the measurement days.

Sleep variables obtained from the accelerometer were sleep latency (SL), which is time to fall asleep in minutes, sleep efficiency (SE), which is the amount of time in which the person is asleep while in bed, the number of minutes of wake time after defined sleep onset (WASO), number of awakenings (AW), time per awakening (TA), and total sleep time (TST). Poor sleep was defined as TST < 6 h or SE ((total sleep time/time in bed) x 100) < 85% [20, 21].

### Statistical analysis

Pearson Product-Moment and Partial Person correlation analysis was used to assess the relationships among SQ, PA, and sedentary behavior, including the average weekly ZC minutes per day, while controlling for age. This approach was chosen because the majority of subjects (78%) were under the age of 23. Additionally, analysis of covariance was employed to compare PA and sedentary behaviors between two groups: those categorized as normal and poor sleepers based on TST and SE criteria as previously defined. Age and daily wear time served as a covariates. Daily wear time was utilized as a covariate to control for any potential influence on zero walking cadence.

## Results

All subjects achieved greater than 10 h of wear time during waking for PA and sedentary analysis (mean wear time ± SD = 16.4 ± 1.9), and there was no difference between males and females. Mean number of days assessed was 7.4 ± 1.3 days, with a range of 4 days to 11 days. Previous research has established an acceptable number of assessment days to be 4 days, including a weekend day, but as little as one day of assessment has been validated [22–24]. There was no association between average daily wear time and the average number of ZC minutes per day (*r* = -0.10, *P* = 0.27). Males were significantly taller and heavier than females. Body mass index (BMI) did not differ between males and females. Males had a shorter mean TST than females, by approximately 25 min. Males had a lower SE than females. No other sleep, PA or sedentary variable differed by sex (Table 1).

### Associations between sleep quality, physical activity, and sedentary behavior

Correlation analysis revealed and association between age and SL (*r* = -0.19, *P* = 0.03), SE (*r* = 0.31, *P* < 0.001), WASO (*r* = -0.32, *P* < 0.001), and AW (*r* = -0.36, *P* < 0.001). Partial correlation analysis revealed that sedentary variables and light intensity PA were associated with sleep quality variables, while MVPA and step counts were not (Table 2). Sedentary minutes per day and number of ZC minutes per day were negatively associated with SE and TST. Additionally, ZC minutes per day was also positively associated with WASO, whereas sedentary minutes per day were not. Light intensity PA minutes were positively associated with SE and negatively associated with WASO and AW.

**Table 2:** Weekly Univariate partial correlations while Controlling for Age. SL = sleep latency, SE = sleep efficiency, TST = total sleep time, WASO = wake after sleep onset (minutes), AW = number of awakenings per night, TA = time per awakening

	SL	SE	TST	WASO	AW	TA
Sedentary min/day	0.02	-0.24*	-0.51**	0.12	0.04	-0.03
0 cadence min/day	-0.01	-0.25*	-0.39**	0.17*	0.11	0.03
Light min/day	-0.03	0.26*	-0.01	-0.32**	-0.33**	-0.09
MVPA min/day	0.01	-0.08	0.01	0.12	0.17	0.08
Steps/day	-0.01	0.04	0.06	-0.02	0.04	0.06

**P < 0.001

*P < 0.05

### Sedentary and physical activity characteristics by sleep quality

To further examine the association with SQ, sedentary behavior and PA, we examined mean differences in sedentary and PA variables by the SQ criteria defined previously. When using TST as the criteria, poor sleepers accumulated more sedentary minutes per day and ZC minutes per day compared to normal sleepers. There were no mean differences in light intensity PA minutes per day, MVPA minutes per day, or steps per day (Table 3). When SE was used as the criteria for poor sleep, poor sleepers accumulated significantly more ZC minutes per day and fewer light intensity PA minutes per day. There was a trend for poor sleepers to accumulate more sedentary minutes per day, but it did not reach statistical significance (Table 4). Other PA variables did not differ.

**Table 3:** Comparison of sedentary behavior variables and physical activity between subjects achieving

	Poor sleep (*n* = 36)	Normal sleep (*n* = 102)	*P* Value
Sedentary time/day (min)	709.6 ± 72.4	649.4 ± 75.5	< 0.001
Zero cadence minutes/day	570.4 ± 77.7	524.5 ± 76.7	< 0.01
Light intensity PA/day (min)	236.7 ± 63.1	235.3 ± 72.8	0.82
MVPA/day (min)	60.6 ± 26.0	59.0 ± 25.7	0.79
Steps/day	9252.6 ± 2929.7	9375.4 ± 3402.9	0.92

< 6 h of total sleep time (Poor Sleep) and those achieving *≥* 6 h of total sleep time (Normal Sleep). Data presented ± SD

PA = physical activity, MVPA = moderate-to-vigorous intensity physical activity

**Table 4:** Comparison of sedentary behavior variables and physical activity between subjects achieving < 85% sleep efficiency (poor sleep) and those achieving *≥* 85% sleep efficiency (normal sleep)

	Poor sleep (*n* = 60)	Normal sleep (*n* = 78)	*P* Value
Sedentary time/day (min)	680.5 ± 73.0	653.3 ± 82.0	0.06
Zero cadence minutes/day	555.9 ± 69.1	521.6 ± 83.7	0.02
Light intensity PA/day (min)	215.1 ± 50.6	251.5 ± 78.8	0.007
MVPA/day (min)	63.0 ± 29.1	56.7 ± 22.5	0.21
Steps/day	9241.9 ± 3580.3	9421.4 ± 3042.9	1.00

Data presented ± SD. PA = physical activity, MVPA = moderate-to-vigorous intensity physical activity

## Discussion

The current study is one of the few studies to examine the frequency of zero step cadence minutes as a marker of sedentary behavior and is the first to examine this marker in relation to sleep quality. Results from the current study show that zero cadence minutes were associated with poor sleep quality to a greater degree than more traditional measures of sedentary behavior. Given the increased ability for the general public to monitor their sleep and PA through consumer wearables, and that step cadence (including zero cadence) is a readily available metric obtained from these wearables, cadence may have utility in evaluating overall health and well-being beyond traditional sedentary behavior assessments. Monitoring and addressing cadence may serve as a valuable tool for improving sleep quality and promoting PA levels in individuals.

Previous studies have examined zero cadence minutes in adults and have established that it is a valid way to quantify sedentary behavior [2, 5, 22]. In U.S. adults, Tudor-Locke et al. reported that, using a sample of 3744 adults ≥20 years, approximately 289 min with zero cadence was accumulated [2]. In contrast to those studies, the current study reported a higher mean value of zero cadence minutes (540). In the current study, wear time was greater when compared to the studies conducted on children [6, 22], which may factor into the difference. Additionally, children are generally more physically active than U.S. adults, resulting in accumulating fewer minutes of zero cadence [6, 24, 25].

When comparing the zero cadence minutes from the current study to that of Tudor-Locke et al. it should be noted that wear time determination was different between the two studies. Tudor-Locke utilized a SAS macro supplied by the National Cancer Institute and non-wear time was defined as ≥ 60 consecutive zeros [2]. In publishing their wear-time algorithm, Choi et al. identified that the method used by Tudor-Locke et al. underestimated wear time, which likely contributed to smaller zero cadence values seen in their study compared to the current study [18].

Previous research examining the relationship between PA, sedentary time, and SQ is limited [7–10, 12, 26]. Kakinami et al. reported an association between sedentary time and poor sleep quality, but no association between PA and sleep quality, similar to findings of the current study [26]. Their study, however relied on subjects means to assess SQ and PA, utilizing the PSQI for SQ, and the International Physical Activity Questionnaire for PA. Further, they assessed sedentary behavior by self-report answers to questions on time spent watching television and computer use, whereas the current study utilized objective means to assess all behaviors.

Liu et al. examined the relationship between SQ and sedentary time in a sample of Japanese college students, and found that an increase in sedentary time (activity < 1.5 METs) was negatively associated with SQ defined by SE [11]. This finding was similar to findings of the current study that also saw a negative association between SE and sedentary time. In contrast, the current study also saw negative associations between sedentary time and TST and saw additional negative associations when ZC was used as the marker for sedentary time. While mean ages were similar between studies, the current study recruited adults 18–65 years, whereas Liu et al. recruited only college students 18–25 years. Their study utilized an ActiGraph device to assess SQ and sedentary time but did not assess PA in any capacity [11].

Two studies utilized accelerometry to assess sedentary time and PA while using the subjective PSQI for their SQ measures [9, 10]. Both studies examined samples of Japanese adults, with mean ages ranging between 52 and 58 years. Kinoshita et al. found that higher sedentary time was negatively associated with SL, but found no association with SE or TST [10]. This is in contrast to findings of the current study that saw no association between sedentary time or ZC to SL, whereas SE and TST were negatively associated with those sedentary variables. Similar to methods of the current study, they further examined mean differences in PA and sedentary time when grouped by sleep quality [10]. Poor sleep, defined as a PSQI global score of 6 or greater, resulted in a significantly greater number of sedentary hours per day, which is similar to findings in the current study. Koohsari et al. reported that replacing 60 min of sedentary time or light-intensity PA with 60 min of MVPA improved SQ in women. The same association was not seen in men [9]. Similarly, the current study did also show an association between light intensity PA minutes and two markers of sleep quality (WASO and AW) but did not show any relationship to MVPA.

To date, three studies have simultaneously assessed SQ, sedentary time and PA through objective means [7, 8, 12]. Kahlhofer et al. utilized the Actiwatch 2 actigraphy watch to assess SQ, and the activPAL™ accelerometer to assess PA and sedentary time in a sample of German college students. The activPAL measures minutes of total sedentary time, as well as time spent lying, sitting, and standing. The activPAL does not use the intensity cutpoints that the ActiGraph devices use, instead reporting steps, METs for intensity. Similar to the current study, they reported no association between PA and SQ, but also no association between SQ and sedentary time. This is in contrast to the current study that found significant associations between ZC minutes, sedentary time per day, and multiple SQ variables [8]. In a sample of older Japanese adults, Seol et al. reported that light-intensity PA was associated with SE, WASO, and AW, and that sedentary time was negatively associated with those SQ variables. Further, they reported that MVPA was negatively associated with AW. The current study saw no association between MVPA and SQ variables but did see a relationship between light-intensity PA, WASO, and AW. Subjects for Seol et al. were significantly older than the current study.

Previous research from our lab examined the relationship between SQ, sedentary time, and PA a sample of 81 college students [7]. For this study, the ActiGraph GT3X accelerometer was used to assess all three. In this sample, no association between PA and SQ was found. However, sedentary minutes per day were negatively associated with TST and AW. Additionally, the number sedentary bouts per day and total time in sedentary bouts per day were also negatively associated with TST [7]. Similar to the current study, our previous study also examined mean differences in sedentary behavior between poor sleepers and normal sleepers and found that poor sleepers (TST < 6 h) accumulated significantly higher number of sedentary bouts and sedentary minutes per day, with no mean difference in any PA variable. The current study also reported that poor sleepers, using TST criteria accumulated higher sedentary minutes per day. The current study, however, incorporating ZC as an additional measure of sedentary time, found that poor sleepers, by TST and SE criteria, accumulated higher number of ZC minutes per day.

The strength of the current study was the use of objectively assessed SQ, PA, step cadence, and sedentary variables with validated and reliable devices and data processing. Our assessment methods are similar to the methods employed by NHANES [13]. Our study is limited by our relatively small sample size when compared to studies using NHANES data. Additionally, a majority of our subjects were younger, while our age range encompassed 18 to 65 years. Adjustments were made in the statistical analysis to account for this.

In conclusion, our study is the first to explore the relationship between PA, sedentary behavior, and sleep quality using zero step cadence as a specific marker of sedentary behavior. Previous research has validated zero step cadence as an effective metric for assessing sedentary time. Our findings indicate that zero cadence might be a more effective measure of sedentary behavior than other variables derived from activity counts measures by accelerometers. Moreover, zero cadence data can be easily collected using a wide range of devices, including those available commercially. This research advances our understanding of the connections between lifestyle factors and health-related outcomes, potentially allowing the general public knowledge and informing healthier lifestyles. Further research is warranted to examine the impact of step cadence on sleep and other health outcomes.

## Data Availability

The data that support the findings of this study are available from the corresponding author upon reasonable request.
